# HOXB9 Expression Correlates with Histological Grade and Prognosis in LSCC

**DOI:** 10.1155/2017/3680305

**Published:** 2017-07-20

**Authors:** Chuanhui Sun, Changsong Han, Peng Wang, Yinji Jin, Yanan Sun, Lingmei Qu

**Affiliations:** ^1^Department of Otorhinolaryngology, Head and Neck Surgery, The Second Affiliated Hospital, Harbin Medical University, Harbin 150086, China; ^2^Department of Pathology, Harbin Medical University, Harbin 150081, China; ^3^Department of Otorhinolaryngology, Head and Neck Surgery, The Fifth Affiliated Hospital, Harbin Medical University, Daqing 163316, China

## Abstract

The purpose of this study was to investigate the HOX gene expression profile in laryngeal squamous cell carcinoma (LSCC) and assess whether some genes are associated with the clinicopathological features and prognosis in LSCC patients. The HOX gene levels were tested by microarray and validated by qRT-PCR in paired cancerous and adjacent noncancerous LSCC tissue samples. The microarray testing data of 39 HOX genes revealed 15 HOX genes that were at least 2-fold upregulated and 2 that were downregulated. After qRT-PCR evaluation, the three most upregulated genes (HOXB9, HOXB13, and HOXD13) were selected for tissue microarray (TMA) analysis. The correlations between the HOXB9, HOXB13, and HOXD13 expression levels and both clinicopathological features and prognosis were analyzed. Three HOX gene expression levels were markedly increased in LSCC tissues compared with adjacent noncancerous tissues (*P* < 0.001). HOXB9 was found to correlate with histological grade (*P* < 0.01) and prognosis (*P* < 0.01) in LSCC. In conclusion, this study revealed that HOXB9, HOXB13, and HOXD13 were upregulated and may play important roles in LSCC. Moreover, HOXB9 may serve as a novel marker of poor prognosis and a potential therapeutic target in LSCC patients.

## 1. Introduction

Laryngeal squamous cell carcinoma (LSCC) is one of the most common forms of highly aggressive cancer and occurs with head and neck malignancies [1]. Although there have been improvements in therapy over the last 20 years, the mortality rate of LSCC remains very high [2]. At present, the detection and prediction of LSCC are primarily based on histopathological classification and TNM staging system [3]. However, some LSCC may be undetectable in some patients due to the limits of these methods. Therefore, effective biomarkers and target genes that are involved in the molecular mechanisms of LSCC need to be identified.

The Homeobox genes were highly conserved during evolution. There are 39 genes belonging to class I Homeobox genes in humans which are named HOX genes and divided into 4 clusters (A–D). Each cluster has 13 paralogous groups [4, 5]. It has been reported that HOX genes play critical roles in normal embryonic development, cell differentiation, and other processes in eukaryotic cell life [6]. Some studies have found that a number of HOX genes play important roles in neoplastic transformation and tumor progression [7, 8]. HOX genes have been found to be aberrantly expressed in many tumors such as breast [9, 10], leukemia [11, 12], lung [13, 14], liver [15], and gastric cancer [16]. However, there is minimal research on the relationships between HOX genes and HNSCC, especially LSCC.

Thus, our present study attempted to identify HOX genes involved in LSCC pathogenesis through a HOX gene expression profile analysis. Upregulated HOX genes were validated by qRT-PCR. The top three HOX genes were chosen for further testing by tissue microarray, and the potential associations of the top three HOX gene levels with clinicopathological features and patients' overall survival (OS) were evaluated.

## 2. Materials and Methods

### 2.1. Collection of Patient Samples

A total of 25 patients subjected to LSCC surgical resection at the Second Affiliated Hospital of Harbin Medical University were recruited in this study. Among them, 5 patients were chosen for profile analysis, and another 20 were used for qRT-PCR validation. Their fresh paired cancerous and adjacent noncancerous tissues were collected and frozen in liquid nitrogen after surgical resection. Overall, 168 LSCC patients who were treated between 2003 and 2011 were recruited for this study, and their paired cancerous and noncancerous tissue blocks were collected from the Department of Pathology of the Second Affiliated Hospital of Harbin Medical University. All of the LSCC patients in our study had the following inclusion criteria: no history of radiotherapy or chemotherapy and a diagnosis of primary squamous cell carcinoma of the larynx. All 168 patients were followed up for at least five years at the Second Affiliated Hospital of Harbin Medical University. Medical records of patients were reviewed for clinical information. Data included age (median age of 64.2 years; range: 41–83 years), anatomical site (supraglottic and glottic), and tumor size. Pathological grade was classified as well differentiated (*n* = 45), moderately differentiated (*n* = 60), and poorly differentiated LSCC (*n* = 63). In addition, 62 patients were diagnosed with lymph node metastasis, and 106 patients had no lymph node metastasis.

Additionally, tumor stage (T-stage) and clinical staging were classified according to the 2002 TNM staging system of the Union for International Cancer Control (UICC). T-stage was done as T1 + T2 (*n* = 101) and T3 + T4 (*n* = 67); clinical staging was done as stage I (*n* = 36), stage II (*n* = 62), stage III (*n* = 38), and stage IV LSCC (*n* = 32). The period of OS was defined as the time from the surgical date to death or last follow-up day. All patients provided written informed consent in accordance with ethical standards of the Declaration of Helsinki. The study protocol was approved by the Ethics Committee of Harbin Medical University.

### 2.2. Microarray Hybridization Experiments

Five human samples (containing paired neoplastic and nonneoplastic margins) were used for the microarray test. This experiment was performed as described previously [17]. Briefly, the total RNA was isolated from laryngeal carcinoma and corresponding adjacent nonneoplastic tissues (100 mg) using TRIzol reagent (Invitrogen, Carlsbad, CA, USA) and quantified using the NanoDrop 1000 (NanoDrop Technologies, Rockland, DE, USA). Agarose gel electrophoresis was used to evaluate the quality of the RNA integrity. Microarray experiments were carried out using CodeLink Whole Genome Bioarrays (GE Healthcare, Piscataway, NJ, USA) and arrays were scanned on a GenePix 4000B Array Scanner (Axon Instruments, USA) according to the recommended scanning procedures and settings. Normalized transcript signals were collected by quantile normalization [18]. To visualize the expression of all 39 HOX genes, hierarchical clustering was applied (MEV4.0, Boston, MA, USA). The differences in HOX gene expression between neoplastic and nonneoplastic samples were calculated by the ratio of the mean normalized fluorescence values. Genes with greater than 2-fold upregulated changes were chosen for further qRT-PCR analysis.

### 2.3. RNA Extraction and qRT-PCR

Total RNA was isolated from the samples of LSCC tissues and noncancerous margins [*n* = 20] using TRIzol reagent (Invitrogen, Carlsbad, CA, USA) according to the manufacturer's instructions. A high capacity RNA-to-cDNA kit (Life Technologies, Paisley, UK) was used to reverse-transcribe total RNA into cDNA. Glyceraldehyde-3-phosphate dehydrogenase (GAPDH) was used as an internal control. The relative target gene mRNA transcript levels compared to GAPDH were measured by qRT-PCR using the 2^−ΔΔCt^ method [19]. The sequences of all primers are provided in supplementary Table  1 (S1 Table) in Supplementary Material available online at https://doi.org/10.1155/2017/3680305.

### 2.4. Tissue Microarray and Immunohistochemistry

A tissue microarray was constructed using 168 tumors and corresponding controls. Two cores from the selected areas were punched out and arrayed in a recipient block. All cancerous and noncancerous tissues were reviewed by two pathologists (Changsong Han/Yinji Jin). Each core was assigned a location chip number linked to the corresponding patient medical data. To evaluate the expression of the top three upregulated HOX genes, immunohistochemical staining was performed by tissue microarray. After the deparaffinization and rehydration of paraffin slides, antigen retrieval was carried out with EDTA (Ethylenediaminetetraacetic Acid, pH 8.0) in a pressure cooker. The sections were washed in PBS (phosphate-buffered saline) and treated with 3% H_2_O_2_ for 8 min. After protein block (10% goat serum), the slides were incubated with primary antibody against HOXB9 (1 : 200, Abcam, Cambridge, MA, USA), HOXB13 (1 : 200, Abcam, Cambridge, MA, USA), and HOXD13 (1 : 100, Abcam, Cambridge, MA, USA) overnight at 4°C. After being washed with PBS, the slides were treated with mouse anti-rabbit secondary IgG antibody at 37°C for 30 min and stained with DAB (diaminobenzidine). The relative levels of HOXB9, HOXB13, and HOXD13 expressions were evaluated as described previously [20]. Briefly, only a percentage of positively stained cells were considered to evaluate the expression. Ten high-power fields (magnification ×400) were chosen randomly by two experienced pathologists (Changsong Han/Yinji Jin). The percentages of positive cancer cells were scored as follows: 0: none; 1: <10%; 2: 10–50%; and 3: >50%. A score of 2 was used to distinguish between low (<2) and high (≥2) levels of HOX gene expression.

### 2.5. Statistical Analysis

The results were analyzed with SAS software version 9.1 (SAS Institute Inc., Cary, NC, USA). The between-group differences in qRT-PCR levels were calculated with one-way ANOVA and least-significant difference (LSD) test. Data were expressed as the mean ± standard deviation (SD). The correlations between HOXB9, HOXB13, and HOXD13 immunohistochemical expression levels and clinicopathological data were determined using Pearson's chi-squared test. The overall survival (OS) of patients was calculated using the Kaplan-Meier survival curve. The results were considered significant only when *P* < 0.05.

## 3. Results

### 3.1. Microarray Analysis of 39 HOX Genes' Expressions in LSCC Tissues

**Figure 1:**
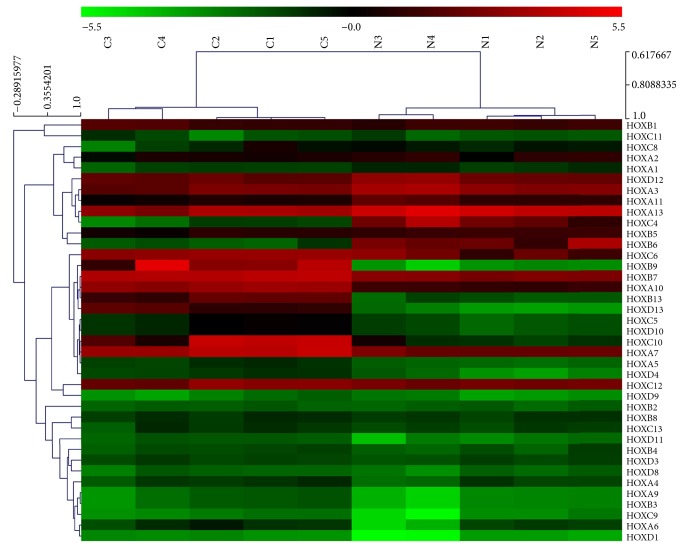
Microarray analysis of all 39 HOX genes in LSCC. Hierarchical clustering is shown as a heat map, and relative gene expression levels are shown in color scales (green, below the mean; red, above the mean; black, median expression). Columns C1–C5 are 5 different LSCC samples, and columns N1–N5 are the 5 corresponding noncancerous tissues (*n* = 5).

To identify potential target genes involved in the molecular mechanisms of LSCC, microarray analysis was carried out.  Figure 1 shows the expression patterns of all 39 HOX genes in cancerous and noncancerous tissues. As shown in the heat map, among the 39 HOX genes, 15 genes were upregulated and 2 genes were downregulated (≥2-fold), which is also summarized in Table 1.

### 3.2. qRT-PCR Validation of Upregulated HOX Genes in LSCC Tissues

**Figure 2:**
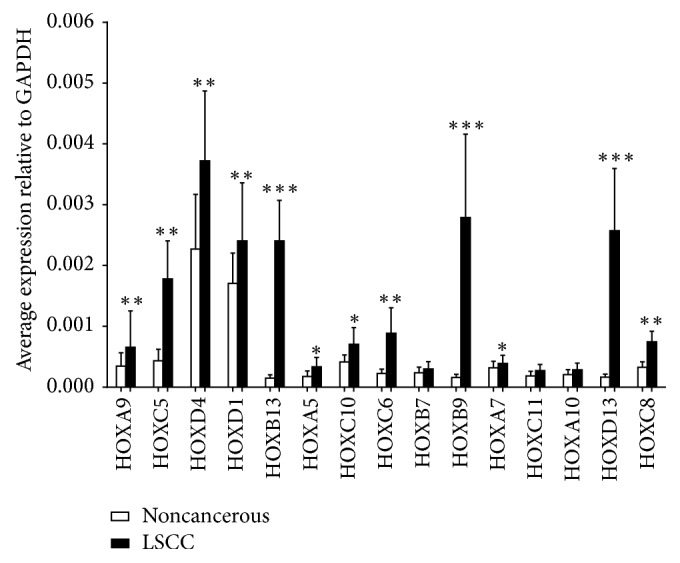
Mean expression of all 15 upregulated HOX genes as tested by RT-PCR in a panel of noncancerous and LSCC tissues. Data are presented relative to the internal endogenous control GAPDH. Among the 15 HOX genes, HOXB9, HOXB13, and HOXD13 show the most significant increase in expression in LSCC compared to noncancerous tissues. ^*∗*^*P* < 0.05, ^*∗∗*^*P* < 0.01, and ^*∗∗∗*^*P* < 0.001 (*n* = 20).

The set of 15 upregulated HOX genes was chosen for further qRT-PCR validation in a larger cohort of patients (*n* = 20). As shown in Figure 2, most of the PCR results were consistent with the microarray analysis. HOXB9, HOXB13, and HOXD13 in particular showed significant differences between LSCC and noncancerous margins (*P* < 0.001).

### 3.3. IHC (Immunohistochemistry) Detection of HOXB9, HOXB13, and HOXD13 in LSCC Tissue Chip

**Figure 3:**
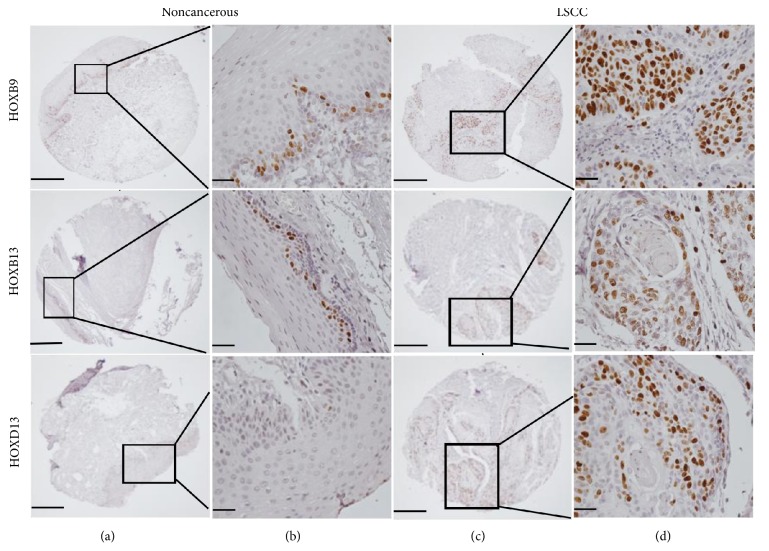
Immunohistochemical analysis of HOXB9, HOXB13, and HOXD13 expressions. The HOXB9, HOXB13, and HOXD13 expressions in LSCC tissues and the corresponding noncancerous tissues were determined by tissue microarray. Rows 1–3 are HOXB9, HOXB13, and HOXD13 expressions, respectively. Columns (a) and (b) are staining in noncancerous tissues, and columns (c) and (d) are staining in LSCC tissues. Images in columns (a) and (c) are under ×40 magnification (bar = 500 *μ*m), and those in columns (b) and (d) are under ×400 magnification (bar = 50 *μ*m) (*n* = 168).

To further evaluate the potential role of the top three differential genes (HOXB9, HOXB13, and HOXD13) in LSCC, more large cohort patients (*n* = 168) were used for TMA analysis. The protein expression levels of HOXB9, HOXB13, and HOXD13 in 168 paired cancerous and adjacent noncancerous tissues were detected by IHC. All three HOX genes exhibited nuclear location. As shown in Figure 3, high levels of HOXB9, HOXB13, and HOXD13 were observed in LSCC tissues (76.2%, 128/168; 67.9%, 114/168; and 70.2%, 118/168, resp.), whereas low levels were found in the noncancerous margins (9.5%, 16/168; 10.7%, 18/168; 13.7%, 23/168, resp.). These results implied that HOXB9, HOXB13, and HOXD13 were overexpressed in LSCC tissues.

### 3.4. Correlation of HOXB9, HOXB13, and HOXD13 Expressions with Clinicopathological Features of LSCC Patients

The main clinicopathological features of the 168 LSCC patients and the correlation of HOXB9, HOXB13, and HOXD13 levels with sample characteristics are shown in Table 2. In general, the statistical results revealed that there was no significant association between the HOXB13 and HOXD13 expression levels and all clinicopathological features. Similarly, there was no significant correlation between HOXB9 expression levels and age, tumor location, and other variables of LSCC patients. However, the level of HOXB9 expression in LSCC patients with a clear moderate pathological grade was significantly higher than that in those with a poorly differentiated grade (*P* = 0.003). Therefore, the HOXB9 expression levels seem to be significantly correlated with histological grade in LSCC.

### 3.5. Relationship between HOXB9, HOXB13, and HOXD13 Expressions and Survival of LSCC Patients

**Figure 4:**
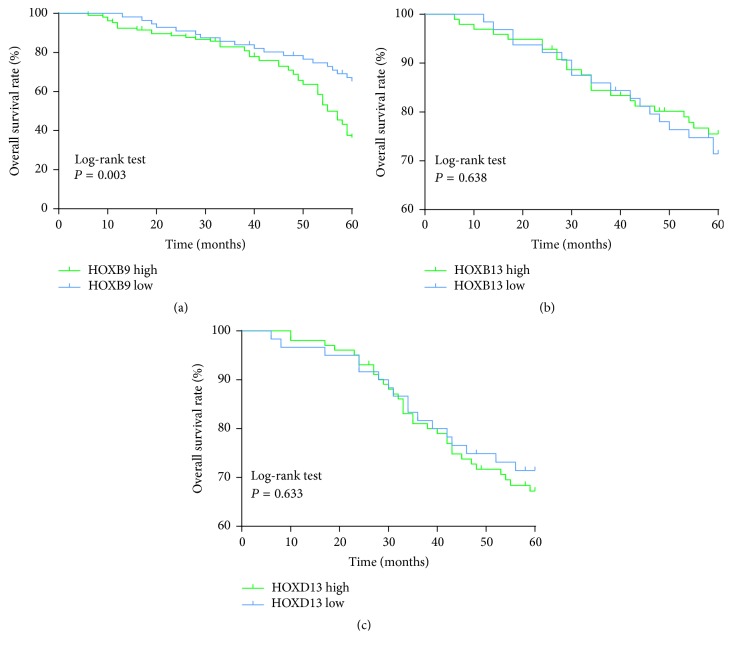
Survival analysis of the correlation of high/low (a) HOXB9, (b) HOXB13, and (c) HOXD13 expressions with overall survival in the patients with LSCC. The overall survival rate was estimated by the Kaplan-Meier method and analyzed by the log-rank test (*n* = 162).

To further evaluate the clinical significance of high HOX gene expression in LSCC, the survival curve was used to compare the difference in survival rate between high and low expression levels of HOXB9, HOXB13, and HOXD13 patients (*n* = 162, 6 patients were lost to follow-up). The results showed that the overexpression of HOXB9 was significantly associated with poor prognosis at 60 months (*P* = 0.003, Figure 4(a)). However, the HOXB13 and HOXD13 expression levels were not correlated with the prognosis of LSCC patients (*P* > 0.05, Figures 4(b) and 4(c)).

## 4. Discussion

In the present study, we report that a set of HOX genes were upregulated in LSCC tissues. Among them, HOXB9, HOXB13, and HOXD13 had the highest expression levels and were further investigated using a prognostic tissue microarray (TMA). We found that HOXB9, HOXB13, and HOXD13 were all overexpressed in LSCC tissues compared with the corresponding adjacent noncancerous tissues. Furthermore, a high expression level of HOXB9 was found to be associated with high histological grade and poor prognosis in LSCC. To the best of our knowledge, the present findings provide the first evidence that high levels of HOXB9 expression may be a valuable marker for the development and prognosis of patients with LSCC.

As a subtype of HNSCC (head and neck squamous cell carcinoma), LSCC is considered as one of the most common malignancies worldwide. The molecular mechanisms of LSCC are still poorly understood, despite recent advances in the treatment of this cancer. The development and progression of LSCC form a complex process that involves interactions among many factors. HOX gene expression is dysregulated in several cancers [9–16]. However, expression of HOX genes varies among different types of cancer. Although some genes were found to act as oncogenes in solid tumors, others showed downregulation in different types of cancer [21] and acted as tumor-suppressor genes [22].

In the present study, the expression of all 39 HOX gene family members was tested using mRNA microarray and then validated by qRT-PCR in LSCC. Because the downregulation of HOX genes occurs via multiple and spatiotemporally controlled mechanisms [23], we did not choose the two downregulated genes (HOXC4 and HOXB6) for further analysis. Of the upregulated genes (15 of the 39 members), HOXB9, HOXB13, and HOXD13 were identified as the top 3 overexpressed HOX genes (fold change > 14). This is partly in keeping with a recent study in HNSCC [24], but that study also showed that other HOX genes were upregulated in LSCC [25]. The discrepancy between these findings may be caused by different severities of LSCC patients and the different methods of preparing samples. We are interested in further study to perform a more detailed analysis in LSCC using HOX gene expression profiles.

For the further validation of our microarray and PCR results, a prognostic TMA with 168 LSCC samples was used for immunohistochemical studies. To the best of our knowledge, our study is the first to evaluate HOX gene expression in LSCC using a prognostic TMA with large cohort tumors. Our finding showed that the top 3 genes (HOXB9, HOXB13, and HOXD13) were all overexpressed and that high HOXB9 expression was significantly associated with a high histological grade and poor prognosis of patients with LSCC. In a recent study similar to ours, deregulation of 13 paralogous HOX genes in oral squamous cell carcinoma (OSCC), which has a similar histological type to LSCC, was observed. Both HOXB13 and HOXD13 showed high nuclear and cytoplasmic expression, and HOXD13 overexpression was inversely related to overall survival [20]. In contrast to the same study, our finding showed that HOXB13 and HOXD13 were overexpressed in LSCC samples. However, we did not detect cytoplasmic staining, and neither HOXB13 nor HOXD13 showed a correlation with clinicopathological features of LSCC patients. However, it is difficult to directly compare our results with those seen in OSCC tissues, given the different genomic background and histological origin. In fact, some HOX genes showed different expression patterns across different tumor types and even across different cell types in the same tumor. HOXB13, for instance, has a controversial role in prostate cancer development because it has been suggested to act both as an oncogene and as a tumor-suppressor gene. A possible explanation may be the fact that the role of HOXB13 seems to depend on the histological type and cellular environment [26, 27]. HOXB13 was also reported to be involved in other solid tumors, including breast cancer, ovarian cancer, skin cancer, and cervical cancer [28–34]. However, the expression pattern and role of HOXB13 in these tumors were still significantly different, depending on the cell type and other environmental factors.

A similar expression pattern was observed regarding HOXD13. As another important member of the 13 paralogous HOX genes, the aberrant expression of HOXD13 has been reported in different tumor types [35–37]. Similar to HOXB13, low HOXD13 expression was reported in pancreatic tumors and may be a marker of prognosis [38]. Another recent report concluded that HOXD13 methylation was a common event in breast cancer and was associated with poor survival in patients [39]. In addition, HOXD13 was implicated in neoplastic transformation, resulting in leukemia [40] and different solid tumors [8, 23]. The same occurs in OSCC [20] and in the present study. Here, both qRT-PCR and TMA showed a significantly higher expression of HOXD13, but there was no correlation to clinic pathological variables and overall survival rate in LSCC patients.

In the present study, HOXB9, another HOX gene, was overexpressed in LSCC and correlated with high tumor grade. Our results also showed that patients with higher HOXB9 expression had a significantly poorer prognosis than those with lower expression. Our conclusion that HOXB9 is an important marker of LSCC development and prognosis echoes the findings of other studies that showed that HOXB9 was frequently overexpressed in many tumors [41–46]. High HOXB9 expression in breast cancer was correlated with high tumor grade and poor prognosis [47]. However, decreased HOXB9 expression has been reported related to poor survival in gastric cancer [48]. These findings suggest that HOXB9 gene function varied in different tumors and usually showed tissue-specific features. However, the underlying mechanism for this remains elusive. In a recent study similar to ours in HNSCC, both HOXB9 and miR-196a were highly expressed, and bioinformatics analysis showed that these may be coexpressed. Furthermore, MAMDC2 was identified as a novel target of miR-196a in HNSCC [24]. Target genes for HOX transcription factors are critical to controlling cell biological behavior. Until now, there were no confirmed specific target genes for HOX genes. There is a big amount of data now supporting the therapeutic potential of inhibiting HOX/PBX dimer formation in cancer. However, the only effective HOX/PBX binding inhibitors are the HXR9 peptide and its derivatives. In a study on breast cancer, the sensitivity of breast cancer cell to killing by HXR9 was shown to be strongly related to the expression of HOXB1 through to HOXB9 [49]. However, it has not been reported in other cancers. Furthermore, potential therapeutic target applications may be more challenging as the minimum reported *K*_*d*_ for PBX binding was 65 *μ*M [50]. Therefore, developing a small molecule inhibitor remains an important clinical goal. Thus, new strategies to eliminate this interaction should be addressed in future research. In addition, to better understand the biological role and functional mechanisms of HOX genes in LSCC, more research should be performed both in vitro and in vivo in the future.

## 5. Conclusions

In conclusion, our study demonstrated the presence of three marked upregulated HOX genes (HOXB9, HOXB13, and HOXD13) in LSCC. A high level of HOXB9 correlated with high histological grade and poor prognosis in LSCC patients. Our findings suggest that HOXB9 may serve both as an oncogene and as a potential marker for the development and prognosis in LSCC.

## Supplementary Material

Supplementary Table 1 (S1 Table): All sequences of primers for 15 upregulated HOX genes.

## Figures and Tables

**Table 1:** Upregulated HOX genes (≥2-fold change) in LSCC samples relative to noncancerous samples tested by microarray analysis.

Gene	Fold change	NCBI access
HOXA9	2.24	NM_152739.3
HOXC5	2.69	NM_018953.2
HOXD4	2.78	NM_014621.2
HOXD1	2.36	NM_024501.1
HOXB13	*14.62*	NM_006361.5
HOXA5	2.05	NM_019102.1
HOXC10	6.19	NM_017409.3
HOXC6	4.42	NM_004503.3
HOXB7	2.22	NM_004502.3
HOXB9	*20.59*	NM_024017.3
HOXA7	2.54	NM_006896.3
HOXC11	2.09	NM_014212.3
HOXA10	4.08	NM_018951.2
HOXD13	*16.47*	NM_000523.3
HOXC8	2.89	NM_022658.3

**Table 2:** Correlation of HOXB9, HOXB13, and HOXD13 genes expression levels with clinicopathological variables in LSCC patients (*n* = 168).

Variable	Cases (*n* = 168)	HOXB9 expression		*P* value	HOXB13 expression		*P* value	HOXD13 expression		*P* value
		High	Low		High	Low		High	Low	
Age (years)										
≤60	75	55	20	0.435	46	29	0.104	55	20	0.431
>60	93	73	20		68	25		63	30	
Tumor location										
Supraglottic	70	50	20	0.221	50	20	0.402	45	25	0.154
Glottic	98	78	20		6	34		73	25	
Tumor size (cm)										
<2.5	72	50	22	0.075	46	26	0.34	52	20	0.691
≥2.5	96	78	18		68	28		66	30	
Pathological grade										
Well/moderate	105	72	33	0.003^*∗*^	70	35	0.67	70	35	0.191
Poor	63	56	7		44	19		48	15	
Lymphatic invasion										
No	106	80	26	0.775	70	36	0.509	72	34	0.466
Yes	62	48	44		44	18		46	16	
T classification										
T1 + T2	101	76	25	0.725	72	29	0.243	70	31	0.746
T3 + T4	67	52	15		42	25		48	19	
Clinical stage										
I + II	98	72	26	0.327	65	33	0.615	68	30	0.776
III + IV	70	56	14		49	21		50	20	

^*∗*^ *P* < 0.01.
