# Supplementary material for: HOXB9 Expression Correlates with Histological Grade and Prognosis in LSCC
Source: Biomed Res Int. 2017 Jul 20;2017:3680305. doi: 10.1155/2017/3680305 (PMC5541786; doi:10.1155/2017/3680305)
Supplement: Supplementary file 1 — Supplementary Table 1 (S1 Table): All sequences of primers for 15 upregulated HOX genes. [file 3680305.f1.docx]

| Gene Forward primer Reverse primer |
| --- |
| HOXA9 TACGTGGACTCGTTCCTGCT CGTCGCCTTGGACTGGAAG  HOXC5 AGAGCCCCAATATCCCTGC CGGTGGGAAAGTGATGCTT  HOXD4 CCCTCCGTGCGAGGAGTAT GAAAGGCTGCTCACCGAAGT  HOXD1 CTTCGGCAACGGCTACTACAG TGACACGTCCATGTACTTCTCC  HOXB13 AGCTCCCGTGCCTTATGGTTA GGCTGGTAGGTTCCCGGATA  HOXA5 AACTCATTTTGCGGTCGCTAT TCCCTGAATTGCTCGCTCAC  HOXC10 ACATGCCCTCGCAATGTAACT GAGAGGTAGGACGGATAGGTG  HOXC6 ACAGACCTCAATCGCTCAGGA AGGGGTAAATCTGGATACTGGC  HOXB7 TTCCCAGAACAAACTTCTTGTGC GCATGTTGAAGGAACTCGGCT  HOXB9 CCATTTCTGGGACGCTTAGCA TGTAAGGGTGGTAGACGGACG  HOXA7 TCGTATTATGTGAACGCGCTT CAAGAAGTCGGCTCGGCATT  HOXC11 ATGTTTAACTCGGTCAACCTGG GCATGTAGTAAGTGCAACTGGG  HOXA10 CTCGCCCATAGACCTGTGG GTTCTGCGCGAAAGAGCAC  HOXD13 CTTCGGCAACGGCTACTACAG TGACACGTCCATGTACTTCTCC  HOXC8 ACCGGCCTATTACGACTGC TGCTGGTAGCCTGAGTTGGA |

S1 Table . A full list of qRT-PCR primers used in this study.
